# Impact of the COVID‐19 Pandemic on Antibiotic Prescribing by Dental Practitioners Across the United Kingdom's Four Countries: A Pharmacoepidemiological Study of Population‐Level Dispensing Data, 2016–2023

**DOI:** 10.1111/cdoe.13037

**Published:** 2025-03-10

**Authors:** Jonathan Bowman‐Newmark, Amin Vahdati, Anup Karki, Linda Young, Gerry Cleary, Wendy Thompson

**Affiliations:** ^1^ Institute of Education in Healthcare and Medical Sciences University of Aberdeen Aberdeen UK; ^2^ Centre for Biostatistics University of Manchester Manchester UK; ^3^ Primary Care Division, Public Health Wales Cardiff UK; ^4^ Dundee Dental Education Centre, NHS Education for Scotland Dundee UK; ^5^ Strategic Planning and Performance Group, Department of Health Derry UK; ^6^ Division of Dentistry University of Manchester Manchester UK

**Keywords:** antibiotic prescribing, antimicrobial stewardship, COVID‐19, dentistry, health services research, pharmacoepidemiological study

## Abstract

**Objectives:**

To evaluate and compare the rates of antibiotic prescribing by dental practitioners across the constituent countries of the United Kingdom between March 2020 and August 2023 and to estimate the total ‘excess’ prescribing that occurred during this interval beyond the rates predicted based upon trends between March 2016 and February 2020.

**Methods:**

Retrospective pharmacoepidemiological study of dental practitioners' antibiotic prescribing, by secondary analysis of population‐level National Health Service dispensing data from England, Scotland, Wales and Health and Social Care dispensing data from Northern Ireland.

**Results:**

Effective August 2023, the antibiotic items dispensed rate for each country remained in excess of that predicted based upon pre‐pandemic trends. Between March 2020 and August 2023, those rates were 175.6, 227.2, 195.0 and 321.8 antibiotic items per 1000 population for England, Scotland, Wales and Northern Ireland, respectively. Those represented estimated total ‘excesses’ of 27.7% (95% confidence limit [CL], 14.8, 43.7), 43.3% (95% CL, 29.9, 60.0), 33.2% (95% CL, 20.4, 49.0) and 42.9% (95% CL, 27.6, 62.3). Pairwise comparisons showed statistically significant differences between England and Scotland, England and Northern Ireland, and Wales and Northern Ireland (*p* < 0.001), Scotland and Wales (*p* = 0.001), and Scotland and Northern Ireland (*p* = 0.009). There was no statistically significant difference between England and Wales.

**Conclusions:**

With shared prescribing guidelines and a single professional regulatory framework, it was unsurprising that similar antibiotic prescribing trends were found across the United Kingdom. Further research is required to investigate the reasons for the differences.

**Abbreviations**
CL: confidence limit
COVID‐19: coronavirus disease‐2019
HSC: Health and Social Care
IQR: interquartile range
LCL: lower confidence limit
MAPE: mean absolute percentage error
MaxAPE: maximum absolute percentage error
NHS: National Health Service
NISRA: Northern Ireland Statistics and Research Agency
NRS: National Records of Scotland
NS: not statistically significant
ONS: Office for National Statistics
SS: statistically significant
UCL: upper confidence limit
UK: United Kingdom

## 1 Introduction

Recognition of the threat to global health posed by antimicrobial resistance has provoked strategic focus on clinicians' antibiotic prescribing practices [1]. The use, misuse and overuse of antibiotics have all put evolutionary pressures on pathogens and commensals, which have driven adaptation through selection of resistant microorganisms [2]. The World Health Organisation has advocated urgent change in the way antibiotics are used in order to conserve their effectiveness and sustain access for all who need them [3].

In March 2020, coronavirus disease‐2019 (COVID‐19) abruptly altered dental care across the United Kingdom (UK). In an unprecedented departure, routine publicly funded and private dental care pathways were indefinitely suspended when restrictions were introduced to protect the public [4, 5, 6, 7]. Dental practitioners were directed to assess and triage patients' acute dental problems remotely; whenever possible, those problems were to be managed with advice, analgesics, or antimicrobials [8]. Constituent countries of the UK adopted different approaches to face‐to‐face provision of emergency dental procedures, where these had been triaged as necessary due to severe or uncontrolled symptoms. Upon referral, those interventions were facilitated by regional centres in England and Scotland, whereas most dental practices remained open in Wales and Northern Ireland albeit with enhanced infection prevention and control policies which restricted the dental care that could be provided [9]. Dental practices in England and Scotland subsequently re‐mobilised their services during June 2020 [10, 11]; however, dentistry was the only healthcare setting in NHS England to experience increased antibiotic prescribing during 2020 [12]. Qualitative research exploring dental practitioners' perspectives about antibiotic prescribing during the COVID‐19 pandemic revealed high levels of frustration in being restricted in their ability to provide treatment to patients with acute dental problems [13, 14, 15].

Several retrospective studies established that antibiotic items prescribed by dental practitioners in England, Scotland and Northern Ireland increased by between 22% and 49% for various discrete intervals during 2020 and 2021, versus pre‐pandemic levels [13, 14, 15, 16]. However, a comprehensive UK‐wide inventory of the impact of the COVID‐19 pandemic on antibiotic prescribing by dental practitioners—including trends from Wales that had not previously been reported—remained outstanding. Therefore, the objectives of the present study were: to evaluate and compare the rates of antibiotic prescribing by dental practitioners across the constituent countries of the UK, between March 2020 and August 2023; and to estimate the total ‘excess’ prescribing that occurred during this interval, beyond the rates predicted based upon trends between March 2016 and February 2020.

## 2 Methods

### 2.1 Study Design and Population

A retrospective pharmacoepidemiological study of dental practitioners' antibiotic prescribing, by secondary analysis of population‐level National Health Service (NHS) dispensing data from England, Scotland, Wales and Health and Social Care (HSC) dispensing data from Northern Ireland. The study population were patients who were prescribed an oral antibiotic by a dental practitioner that was subsequently dispensed by a community pharmacy between March 2016 and August 2023. The starting point selected coincided with the first complete calendar year of published prescribing data available from Scotland, which ultimately determined the earliest comparisons possible between the four constituent countries of the UK. The exposure was the COVID‐19 pandemic and the associated restrictions that were enacted to protect the public from the effects of its person‐to‐person transmission. Predictive models were applied to estimate ‘excess’ antibiotic items prescribed since March 2020, beyond those forecasted based on pre‐pandemic trends.

### 2.2 Data Sources and Extraction

Study data were obtained from public sector databases of anonymised routinely collected health information not linked to other datasets. Those databases recorded information about antibiotic items prescribed by dental practitioners on NHS or HSC prescription forms that were subsequently dispensed by community pharmacies. In the present study, antibiotics dispensed were used as a surrogate measure for antibiotics prescribed.

Dispensing data from England were sourced from the NHS Business Services Authority, obtained following three separate requests made under the Freedom of Information Act 2000 (ePACT2, NHS Business Services Authority copyright 2022 and 2023). Dispensing data from Scotland were sourced from Public Health Scotland, obtained from published monthly prescribing data for March 2016 through August 2023 (adapted from public sector information licensed under the Open Government Licence v3.0). Dispensing data from Wales were sourced from the NHS Wales Shared Services Partnership, obtained following a request made under the Freedom of Information Act 2000 (ePACT2, NHS Business Services Authority copyright 2023), in combination with published monthly prescribing data for April 2019 through August 2023 (adapted from public sector information licensed under the Open Government Licence v3.0). Dispensing data from Northern Ireland were sourced directly from the Business Services Organisation, obtained from the Family Practitioner Services Pharmacy Payment System (HSC Business Services Organisation copyright).

Monthly total counts of antibiotic items dispensed were compiled for England, Scotland, Wales and Northern Ireland (Table S1). Antibiotic items were defined according to the oral antibacterial drugs listed in the Dental Practitioners' Formulary [17]. Both proprietary and non‐proprietary formulations were included. Doxycycline hyclate 20 mg formulations—indicated solely as an inhibitor of collagenase activity and prescribed exclusively as an adjunctive periodontal therapy—were excluded. The number of antibiotic items counted represented the number of times antibiotics appeared on prescription forms, which remained distinct from the number of doses of antibiotics prescribed. Mid‐year population estimates and projections were obtained from the Office for National Statistics, National Records of Scotland and Northern Ireland Statistics and Research Agency (public sector information licensed under the Open Government Licence v3.0) (Table S2).

### 2.3 Outcome Measure

A recognised antibiotic use metric was selected from an international consensus of outpatient quantity metrics [18]. In the present study, this was expressed as the crude event rate ‘antibiotic items dispensed per 1000 population’, which established a stable denominator that adjusted for differences in population sizes between countries over time, weighted by mid‐year population estimate or projection.

### 2.4 Data Analysis

Monthly total counts during the 90‐month study period were dichotomised into a pre‐pandemic time series, between March 2016 and February 2020 (48 months), and a pandemic time series, between March 2020 and August 2023 (42 months). Antibiotic items dispensed per 1000 population since March 2020 were compared against individually specified damped trend predictive models for each of the UK's constituent countries, which were modelled upon their respective pre‐pandemic time series. The modelling framework was selected following a visual inspection of plots; univariate time series data exhibited linear trend characteristics that could reasonably be expected to attenuate during the forecast horizon. No additional transformations were undertaken, and no seasonality was assumed. Each model incorporated level, trend and damping parameters that were optimised by Time Series Modeller (IBM SPSS Statistics for Mac, v27.0; IBM Corporation, Armonk, USA). The predictive models demonstrated mean and maximum absolute percentage errors of 3.2%–4.0% and 9.9%–11.5%, respectively (Table S3).

Estimated ‘excess’ antibiotic items dispensed per month per 1000 population since March 2020 were calculated as the difference between observed and predicted (±95% confidence limits) pandemic time series values, and distributional differences were evaluated for statistical significance. In order to explore differences between the constituent countries of the UK, estimated ‘excess’ antibiotic items dispensed per month per 1000 population for England, Scotland, Wales and Northern Ireland were compared, and distributional differences were evaluated for statistical significance.

Data were analysed using SPSS Statistics (IBM SPSS Statistics for Mac, v27.0; IBM Corporation) and summarised using Excel (Microsoft Excel for Mac, v16.78; Microsoft Corporation, Redmond, USA). Continuous data were assessed for normality, and descriptive statistics were presented according to distribution. Inferential statistics included paired samples *t*‐tests for parametric data and Wilcoxon signed‐ranks tests or the Kruskal–Wallis test with post hoc Dunn's tests for non‐parametric data. The level for statistical significance was *α* < 0.05.

## 3 Results

**FIGURE 1:**
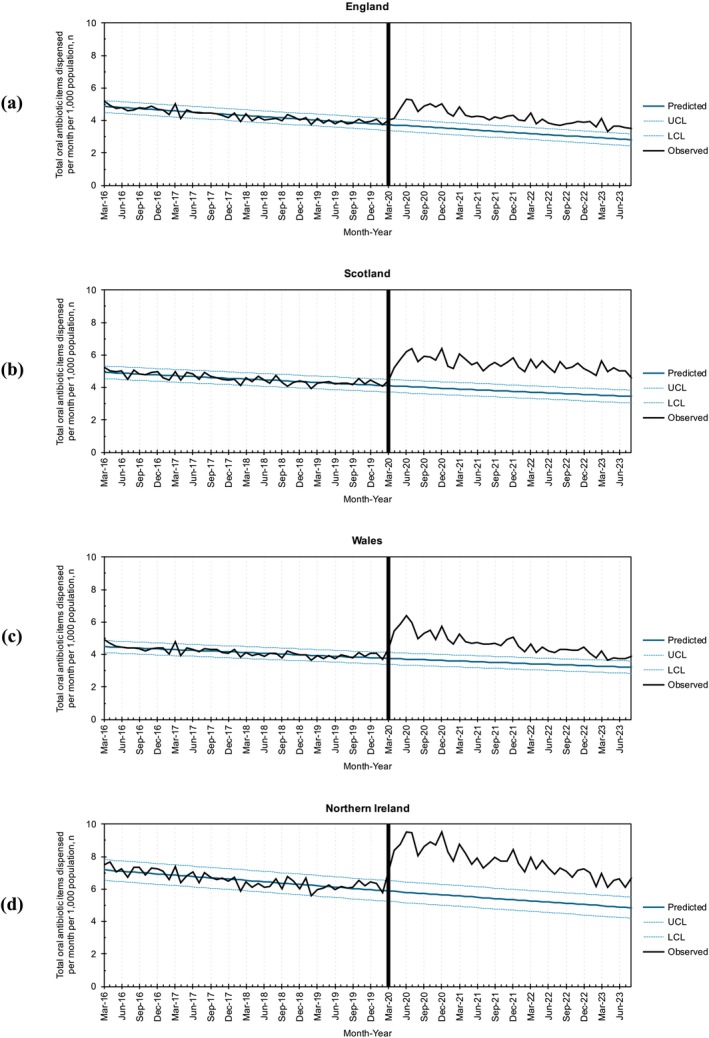
Total oral antibiotic items dispensed per month per 1000 population (observed vs. predictive model) between March 2016 and August 2023: (a) England; (b) Scotland; (c) Wales and (d) Northern Ireland. Observed counts represented by solid black lines and predicted counts represented by solid blue lines (95% upper and lower confidence limits represented by dashed blue lines). LCL, lower confidence limit; UCL, upper confidence limit.

A total of 26 339 560 antibiotic items prescribed by dental practitioners were dispensed by community pharmacies between March 2016 and August 2023. Antibiotic items dispensed per month per 1000 population exhibited dominant downward trends for each of the UK's constituent countries prior to March 2020 (Figure 1). Abrupt increases occurred following March 2020, which coincided with the enaction of COVID‐19 restrictions. Antibiotic items dispensed rates peaked between June and December 2020, and thereafter exhibited predominantly downward trends that varied by country. Effective as of August 2023, the antibiotic items dispensed rate for each country remained in excess of the rate predicted based on pre‐pandemic trends.

**FIGURE 2:**
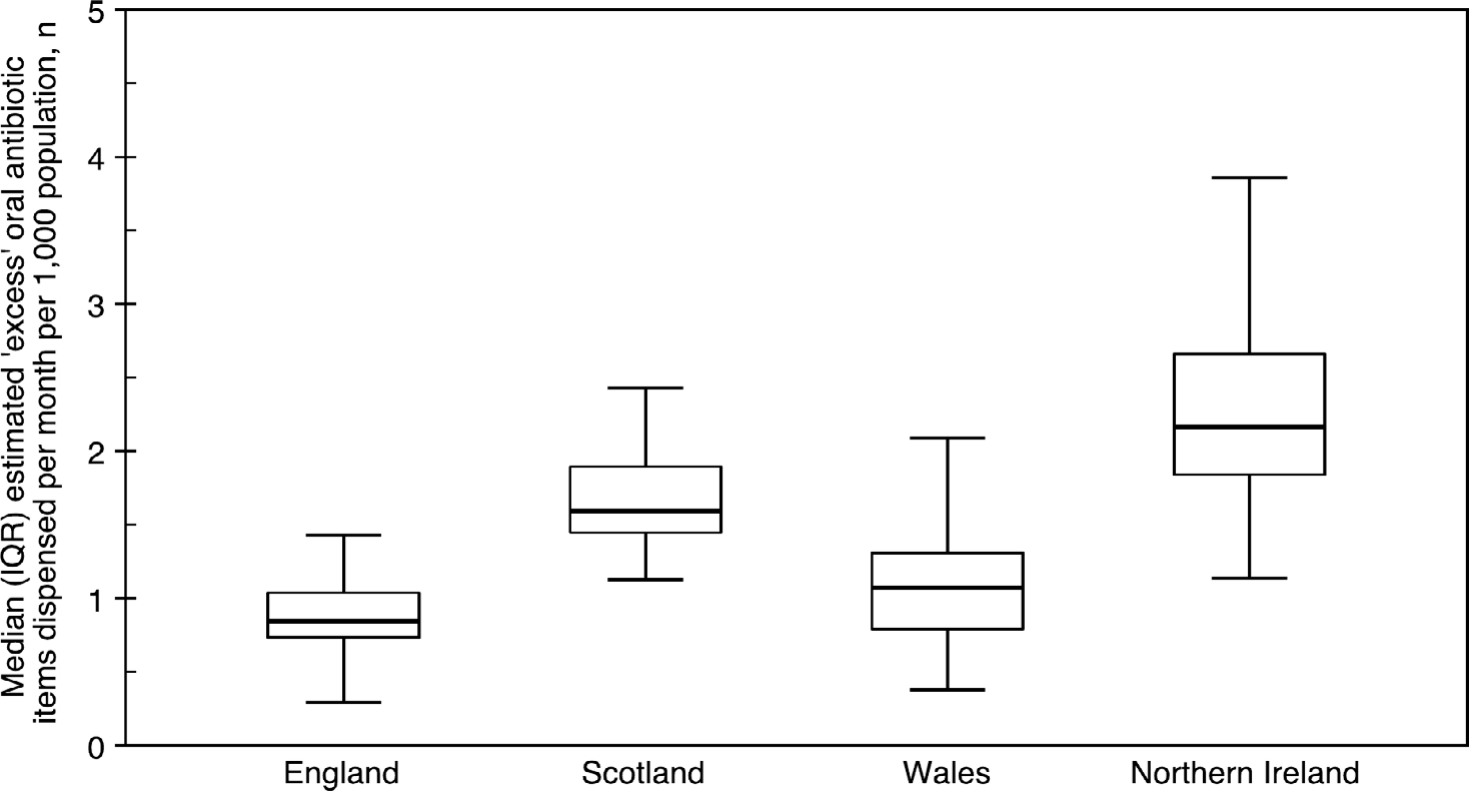
Box and whisker plot comparing median estimated ‘excess’ oral antibiotic items dispensed per month per 1000 population (observed vs. predictive model) between March 2020 and August 2023 for England, Scotland, Wales and Northern Ireland. IQR, interquartile range.

Statistically significant differences between observed and predicted antibiotic items dispensed since March 2020 existed for all constituent countries of the UK (Table 1). A total of 175.6, 227.2, 195.0 and 321.8 antibiotic items per 1000 population were dispensed between March 2020 and August 2023 for England, Scotland, Wales and Northern Ireland, respectively. These represented estimated total ‘excesses’ of 27.7%, 43.3%, 33.2% and 42.9%. There was very strong evidence of a statistically significant difference (Kruskal–Wallis test, *χ* ^2^(3) = 92.3, *p* < 0.001) in the distributions of estimated ‘excess’ antibiotic items dispensed per month per 1000 population across all constituent countries of the UK. The median (interquartile range) estimated ‘excess’ antibiotics dispensed per month per 1000 population were 0.8 (0.7, 1.0), 1.6 (1.4, 1.9), 1.1 (0.8, 1.3) and 2.2 (1.8, 2.7) for England, Scotland, Wales and Northern Ireland, respectively (Figure 2). Post hoc pairwise comparisons provided very strong evidence of a statistically significant difference between England and Scotland, England and Northern Ireland, and Wales and Northern Ireland; and strong evidence of a statistically significant difference between Scotland and Wales and Scotland and Northern Ireland (Table 2). There was no evidence of a statistically significant difference between England and Wales.

**TABLE 1:** Total estimated ‘excess’ oral antibiotic items dispensed per 1000 population (observed vs. predictive model) between March 2020 and August 2023 for England, Scotland, Wales and Northern Ireland.

Country	Total oral antibiotic items dispensed per 1000 population between March 2020 and August 2023			
	Observed, *n*	Predicted, *n* (95% CL)	Estimated ‘excess’, *n* (95% CL)	Estimated ‘excess’, % (95% CL)
England	175.6	137.6 (122.2, 153.0)	38.1 (22.7, 53.4)^a^	27.7 (14.8, 43.7)
Scotland	227.2	158.5 (142.0, 174.9)	68.7 (52.2, 85.1)^b^	43.3 (29.9, 60.0)
Wales	195.0	146.4 (130.9, 161.9)	48.6 (33.1, 64.1)^a^	33.2 (20.4, 49.0)
Northern Ireland	321.8	225.2 (198.3, 252.1)	96.6 (69.7, 123.5)^b^	42.9 (27.6, 62.3)

Abbreviation: CL, confidence limit.

^a^ Statistically significant (Wilcoxon signed‐ranks test, *p* < 0.001).

^b^ Statistically significant (paired samples *t*‐test, *p* < 0.001).

**TABLE 2:** Pairwise comparisons between England, Scotland, Wales and Northern Ireland of estimated ‘excess’ oral antibiotic items dispensed per month per 1000 population (observed vs. predictive model) between March 2020 and August 2023.

Pairwise comparison	Adjusted significance^a^
England—Scotland	< 0.001^SS^
England—Wales	0.304^NS^
England—Northern Ireland	< 0.001^SS^
Scotland—Wales	0.001^SS^
Scotland—Northern Ireland	0.009^SS^
Wales—Northern Ireland	< 0.001^SS^

Abbreviations: NS, not statistically significant; SS, statistically significant.

^a^ Post hoc Dunn's test adjusted using the Bonferroni correction for multiple tests.

## 4 Discussion

Prior to March 2020, antibiotic prescribing by dental practitioners had steadily decreased across the UK. The abrupt changes that transpired following March 2020 provided temporal evidence of expected causal effects from the COVID‐19 pandemic, including restrictions on the provision of dental treatment. Findings that the estimated total ‘excess’ antibiotic items dispensed between March 2020 and August 2023 ranged between 27.7% and 43.3% appeared consistent with previously published reports. Antibiotic items prescribed by dental practitioners in England, Scotland and Northern Ireland increased by 22%–49% for various discrete intervals during 2020 and 2021 [13, 14, 15, 16]. Comparator data from Wales had not previously been described in the peer‐reviewed literature, and the present study reported these data for the first time. The findings that, effective August 2023, the antibiotic items dispensed rate for each country remained in excess of that predicted based upon pre‐pandemic trends highlighted a need for continued monitoring. Additional research is indicated to understand how the rates of antibiotic prescribing relate to face‐to‐face provision of dental procedures. This will necessitate the development of novel methods to indirectly compare measures of publicly funded dental activity across each of the UK's countries.

As a unitary sovereign state, the UK presented an opportunity to explore differences between four countries where dental practitioners' antibiotic prescribing occurred within a single professional regulatory framework [19], where prescribing guidelines were shared [17, 20, 21], and where antimicrobial stewardship continues to be directed by unified national action plans [22, 23]. A key distinction between the countries was that publicly funded dental care provision remained a devolved matter for Scotland, Wales and Northern Ireland. NHS general dental services in England and Wales were contracted based on units of dental activity: patients' payments were banded, dependent upon the complexity of the courses of treatment provided [24, 25]. In contrast, NHS Scotland and HSC in Northern Ireland contracted their general dental services based on fee‐for‐service models; in those countries, patients' payments were 80% of treatment costs and total costs were capped per course of treatment [26, 27]. The similarities in the contracting of NHS general dental services in England and Wales may potentially explain the absence of a significant difference between those countries antibiotic prescribing rates. Contract terms for publicly funded general dental services, and their anticipated impacts upon working environments, may have contributed to the differences in antibiotic prescribing rates found between the constituent counties of the UK. Further research to investigate this hypothesis should inform NHS and HSC dental contract reform to ensure that the way dentistry is incentivised better supports delivery of the UK's national action plan on tackling antimicrobial resistance to realise its vision to contain and control antimicrobial resistance by 2040 [23, 28].

The crucial strength of this study was its representativeness, as data about antibiotic items prescribed by dental practitioners on NHS or HSC prescription forms represented a population rather than a sample. It constituted the most complete geographic coverage reported to date from the UK. Notwithstanding, it is acknowledged that dispensing data may have incompletely reflected the totality of antibiotic prescribing, as some patients may not have taken their prescriptions to a community pharmacy to be dispensed for various reasons, such as delayed prescribing or on account of financial burden [29, 30]. However, non‐dispensing due to financial burden was not expected in Scotland, Wales and Northern Ireland, where prescription charges were abolished in 2011, 2007 and 2010, respectively [31, 32, 33]. Furthermore, eligible patient groups were exempt from prescription costs in England [34]. The present study will also underestimate antibiotic prescribing because dispensing data from community pharmacies did not include items dispensed in hospitals and prisons, or items that were prescribed privately by dental practitioners. This latter category in particular was anticipated to have made an important contribution to antibiotic prescribing in the UK. Nevertheless, its influence was difficult to quantify due to the absence of publicly available databases that compiled antibiotic items prescribed privately by dental practitioners. Therefore, while the findings of this study were considered representative of publicly funded dental services in the UK, further research is required to understand whether they are generalisable to dental practitioners who provide care to patients who pay privately.

An additional methodological strength of this study was its modelling framework. Reliable forecasting relies upon judicious selection of predictive models [35]. Damped trend modelling has been shown to successfully adjust for over‐forecasting when the forecast horizon is substantial [36]. As damped trends eventually level off to become relatively stationary, they typically yield relatively conservative predictions. Mean absolute percentage error, which was calculated in this study as the mean absolute difference between pre‐pandemic time series predicted and observed values, was considered an important measure of model performance. Errors of < 10% have been considered to represent highly accurate forecasting [37]. The errors calculated for each model, between 3.2% and 4.0%, were very small. Moreover, the maximum absolute percentage error across all models was between 9.9% and 11.5%. The small magnitude of those errors provided empirical support for the selection of the modelling framework.

An acknowledged limitation of this study was its inability to evaluate the appropriateness of antibiotic items that were prescribed. Previous studies—undertaken in Wales and Northern Ireland, respectively—had determined that 81% and 52% of antibiotics prescribed during emergency dental appointments were inappropriate [38, 39]. The present study's findings showed that antibiotic dispensing rates were only just returning to pre‐pandemic levels by the end of the study period across the UK's countries, which suggested that high levels of inappropriate prescribing had persisted. According to the core outcome set for dental antibiotic stewardship, appropriateness is an important measure of the quality of antibiotic prescribing; notwithstanding, assessments are challenging to operationalise [40]. The UK Health Security Agency and National Institute for Health and Care Excellence have both recognised that electronic information management systems do not collect data routinely that relates to prescribing within dentistry [12, 41]. The latter body has recommended the implementation of ‘electronic prescribing systems that link indication with the antimicrobial prescription’ [41]. This issue was highlighted in a recent systematic review of dental antibiotic stewardship interventions [42], which included findings reported in relation to an audit and feedback undertaken in Scotland [43]. Further work is needed to operationalise the core outcome set for dental antibiotic stewardship in order to more effectively monitor the quality of dental antibiotic prescribing in the UK.

As the dispensing data was obtained from databases that record information primarily for financial reimbursement purposes, there were no demographic or individual‐level covariate data available (e.g., comorbidities or deprivation status) that would enable further analyses to explain the UK‐wide population trends observed. Analogously, this study was unable to differentiate between antibiotics that were prescribed prophylactically versus therapeutically. Nevertheless, the proportion of antibiotics prescribed as prophylaxis for dental procedures was anticipated to make only a relatively modest overall contribution on account of the particular circumstances and the specific prescribing guidance adopted in the UK [44].

## 5 Conclusion

Substantial ‘excesses’ in antibiotic prescribing by dental practitioners were estimated to have occurred across the UK between March 2020 and August 2023. With shared prescribing guidelines and a single professional regulatory framework, it was unsurprising that similar prescribing trends were found. Further research is required to investigate the hypothesis that significant distributional differences between constituent countries' estimated ‘excesses’ were associated with country‐specific contract terms for publicly funded dental services.

## Author Contributions

Conceptualisation: J.B.‐N. and W.T.; methodology: J.B.‐N. and W.T.; investigation: J.B.‐N., A.V., A.K., L.Y., G.C., and W.T.; data curation: J.B.‐N. and W.T.; formal analysis: J.B.‐N. and W.T.; writing – original draft: J.B.‐N., A.V., G.C., and W.T.; visualisation: J.B.‐N.; writing – review and editing: J.B.‐N., A.V., A.K., L.Y., G.C., and W.T.; supervision: W.T. All authors have read and agreed to the published version of this manuscript.

## Disclosure

W.T. was supported during this work by a clinical lectureship from the National Institute for Health and Care Research.

## Ethics Statement

The authors have nothing to report.

## Conflicts of Interest

The authors declare no conflicts of interest.

## Supporting information


Tables S1–S3.
**Table S1** Total oral antibiotic items dispensed per month for England, Scotland, Wales and Northern Ireland between March 2016 and August 2023.
**Table S2** Mid‐year population estimates or population projections for England, Scotland, Wales and Northern Ireland between 2016 and 2023.
**Table S3** Performance of predictive models for England, Scotland, Wales and Northern Ireland between March 2016 and February 2020 assessed using goodness‐of‐fit criteria.

## Data Availability

Analyses that support the findings of this study are available from the corresponding author upon reasonable request. Dispensing data not already in the public domain were obtained following requests made under the Freedom of Information Act 2000, and were available from the NHS Business Services Authority (FoI‐01142, FoI‐01596 and FoI‐27 646) and NHS Wales Shared Services Partnership (6‐23). Under the terms of data use agreements, it is not possible to reproduce copyright material; however, data may be obtained upon request to the NHS Business Services Authority, NHS Wales Shared Services Partnership and HSC Business Services Organisation.
