# Supplementary material for: Impact of the COVID‐19 Pandemic on Antibiotic Prescribing by Dental Practitioners Across the United Kingdom's Four Countries: A Pharmacoepidemiological Study of Population‐Level Dispensing Data, 2016–2023
Source: Community Dent Oral Epidemiol. 2025 Mar 10;53(4):354–61. doi: 10.1111/cdoe.13037 (PMC12238736; doi:10.1111/cdoe.13037)
Supplement: Supplementary file 1 — Tables S1–S3. Table S1 Total oral antibiotic items dispensed per month for England, Scotland, Wales and Northern Ireland between March 2016 and August 2023. Table S2 Mid‐year population estimates or population projections for England, Scotland, Wales and Northern Ireland between 2016 and 2023. Table S3 Performance of predictive models for England, Scotland, Wales and Northern Ireland between March 2016 and February 2020 assessed using goodness‐of‐fit criteria. [file CDOE-53-354-s001.docx]

**Table S1** Total oral antibiotic items dispensed per month for England, Scotland, Wales, and Northern Ireland between March 2016 and August 2023.

|  | | **Total antibiotic items dispensed, n** | | | |
| --- | --- | --- | --- | --- | --- |
| **Month** | **Year** | **England** | **Scotland** | **Wales** | **Northern**  **Ireland** |
| March | 2016 | 287,309 ^a^ | 28,394 ^b^ | 15,125 ^c^ | 13,952 ^d^ |
| April | 2016 | 272,480 ^a^ | 27,128 ^b^ | 14,370 ^c^ | 14,336 ^d^ |
| May | 2016 | 261,693 ^a^ | 26,888 ^b^ | 13,805 ^c^ | 13,126 ^d^ |
| June | 2016 | 264,380 ^a^ | 27,087 ^b^ | 13,769 ^c^ | 13,494 ^d^ |
| July | 2016 | 255,251 ^a^ | 24,341 ^b^ | 13,507 ^c^ | 12,514 ^d^ |
| August | 2016 | 257,400 ^a^ | 27,361 ^b^ | 13,564 ^c^ | 13,705 ^d^ |
| September | 2016 | 264,799 ^a^ | 26,087 ^b^ | 13,474 ^c^ | 13,672 ^d^ |
| October | 2016 | 261,733 ^a^ | 25,874 ^b^ | 12,938 ^c^ | 12,840 ^d^ |
| November | 2016 | 269,989 ^a^ | 26,557 ^b^ | 13,394 ^c^ | 13,574 ^d^ |
| December | 2016 | 260,437 ^a^ | 26,844 ^b^ | 13,627 ^c^ | 13,555 ^d^ |
| January | 2017 | 259,372 ^a^ | 25,032 ^b^ | 13,586 ^c^ | 13,308 ^d^ |
| February | 2017 | 242,611 ^a^ | 24,112 ^b^ | 12,393 ^c^ | 12,378 ^d^ |
| March | 2017 | 278,285 ^a^ | 27,087 ^b^ | 14,704 ^c^ | 13,862 ^d^ |
| April | 2017 | 229,005 ^a^ | 24,229 ^b^ | 12,109 ^c^ | 12,031 ^d^ |
| May | 2017 | 258,117 ^a^ | 26,636 ^b^ | 13,555 ^c^ | 12,807 ^d^ |
| June | 2017 | 249,266 ^a^ | 26,258 ^b^ | 13,226 ^c^ | 13,261 ^d^ |
| July | 2017 | 247,675 ^a^ | 24,454 ^b^ | 12,927 ^c^ | 12,006 ^d^ |
| August | 2017 | 246,995 ^a^ | 26,653 ^b^ | 13,485 ^c^ | 13,156 ^d^ |
| September | 2017 | 247,850 ^a^ | 25,368 ^b^ | 13,321 ^c^ | 12,652 ^d^ |
| October | 2017 | 246,142 ^a^ | 24,986 ^b^ | 13,247 ^c^ | 12,336 ^d^ |
| November | 2017 | 240,487 ^a^ | 24,468 ^b^ | 12,755 ^c^ | 12,498 ^d^ |
| December | 2017 | 231,494 ^a^ | 24,228 ^b^ | 12,520 ^c^ | 12,146 ^d^ |
| January | 2018 | 249,301 ^a^ | 24,527 ^b^ | 13,247 ^c^ | 12,704 ^d^ |
| February | 2018 | 220,863 ^a^ | 22,374 ^b^ | 11,836 ^c^ | 11,096 ^d^ |
| March | 2018 | 245,382 ^a^ | 24,918 ^b^ | 12,771 ^c^ | 12,113 ^d^ |
| April | 2018 | 221,745 ^a^ | 23,807 ^b^ | 12,075 ^c^ | 11,507 ^d^ |
| May | 2018 | 235,592 ^a^ | 25,601 ^b^ | 12,361 ^c^ | 11,974 ^d^ |
| June | 2018 | 224,739 ^a^ | 24,191 ^b^ | 12,060 ^c^ | 11,525 ^d^ |
| July | 2018 | 226,922 ^a^ | 23,307 ^b^ | 12,555 ^c^ | 11,620 ^d^ |
| August | 2018 | 229,429 ^a^ | 25,884 ^b^ | 12,628 ^c^ | 12,494 ^d^ |
| September | 2018 | 222,538 ^a^ | 23,387 ^b^ | 11,643 ^c^ | 11,385 ^d^ |

(*continued on next page*)

**Table S1** (continued)

|  | | **Total antibiotic items dispensed, n** | | | |
| --- | --- | --- | --- | --- | --- |
| **Month** | **Year** | **England** | **Scotland** | **Wales** | **Northern**  **Ireland** |
| October | 2018 | 244,113 ^a^ | 22,252 ^b^ | 13,053 ^c^ | 12,787 ^d^ |
| November | 2018 | 234,989 ^a^ | 23,528 ^b^ | 12,590 ^c^ | 12,235 ^d^ |
| December | 2018 | 224,138 ^a^ | 23,935 ^b^ | 12,240 ^c^ | 11,361 ^d^ |
| January | 2019 | 235,060 ^a^ | 23,445 ^b^ | 12,359 ^c^ | 12,668 ^d^ |
| February | 2019 | 210,053 ^a^ | 21,622 ^b^ | 11,311 ^c^ | 10,651 ^d^ |
| March | 2019 | 230,827 ^a^ | 23,188 ^b^ | 12,178 ^c^ | 11,332 ^d^ |
| April | 2019 | 215,415 ^a^ | 23,664 ^b^ | 11,612 ^e^ | 11,510 ^d^ |
| May | 2019 | 226,188 ^a^ | 23,947 ^b^ | 12,098 ^e^ | 11,890 ^d^ |
| June | 2019 | 212,729 ^a^ | 22,926 ^b^ | 11,606 ^e^ | 11,335 ^d^ |
| July | 2019 | 224,661 ^a^ | 23,276 ^b^ | 12,246 ^e^ | 11,647 ^d^ |
| August | 2019 | 212,651 ^a^ | 23,350 ^b^ | 11,955 ^e^ | 11,548 ^d^ |
| September | 2019 | 214,955 ^a^ | 22,847 ^b^ | 11,709 ^e^ | 11,626 ^d^ |
| October | 2019 | 229,182 ^a^ | 24,776 ^b^ | 12,714 ^e^ | 12,373 ^d^ |
| November | 2019 | 218,988 ^a^ | 23,127 ^b^ | 11,997 ^e^ | 11,758 ^d^ |
| December | 2019 | 220,740 ^a^ | 24,218 ^b^ | 12,569 ^e^ | 12,189 ^d^ |
| January | 2020 | 229,079 ^a^ | 23,448 ^b^ | 12,689 ^e^ | 12,052 ^d^ |
| February | 2020 | 210,567 ^a^ | 22,256 ^b^ | 11,474 ^e^ | 11,019 ^d^ |
| March | 2020 | 227,236 ^a^ | 24,369 ^b^ | 13,644 ^e^ | 13,612 ^d^ |
| April | 2020 | 232,663 ^a^ | 28,523 ^b^ | 16,957 ^e^ | 15,961 ^d^ |
| May | 2020 | 267,719 ^a^ | 31,463 ^b^ | 18,239 ^e^ | 16,683 ^d^ |
| June | 2020 | 298,900 ^a^ | 33,800 ^b^ | 19,903 ^e^ | 18,046 ^d^ |
| July | 2020 | 296,204 ^a^ | 35,011 ^b^ | 18,531 ^e^ | 17,955 ^d^ |
| August | 2020 | 257,079 ^a^ | 30,636 ^b^ | 15,424 ^e^ | 15,274 ^d^ |
| September | 2020 | 275,461 ^a^ | 32,399 ^b^ | 16,461 ^e^ | 16,387 ^d^ |
| October | 2020 | 282,215 ^a^ | 31,995 ^b^ | 17,096 ^e^ | 16,958 ^d^ |
| November | 2020 | 271,627 ^a^ | 31,114 ^b^ | 15,340 ^e^ | 16,601 ^d^ |
| December | 2020 | 283,882 ^a^ | 34,929 ^b^ | 17,776 ^e^ | 18,065 ^d^ |
| January | 2021 | 251,874 ^a^ | 29,012 ^b^ | 15,326 ^e^ | 15,782 ^d^ |
| February | 2021 | 240,300 ^a^ | 28,348 ^b^ | 14,414 ^e^ | 14,691 ^d^ |
| March | 2021 | 272,301 ^a^ | 33,274 ^b^ | 16,262 ^e^ | 16,656 ^d^ |
| April | 2021 | 244,282 ^a^ | 31,517 ^b^ | 14,861 ^e^ | 15,626 ^d^ |
| May | 2021 | 239,428 ^a^ | 29,695 ^b^ | 14,509 ^e^ | 14,378 ^d^ |

(*continued on next page*)

**Table S1** (continued)

|  | | **Total antibiotic items dispensed, n** | | | |
| --- | --- | --- | --- | --- | --- |
| **Month** | **Year** | **England** | **Scotland** | **Wales** | **Northern**  **Ireland** |
| June | 2021 | 241,887 ^a^ | 30,320 ^b^ | 14,766 ^e^ | 15,084 ^d^ |
| July | 2021 | 237,634 ^a^ | 27,525 ^b^ | 14,411 ^e^ | 13,926 ^d^ |
| August | 2021 | 226,777 ^a^ | 29,294 ^b^ | 14,393 ^e^ | 14,484 ^d^ |
| September | 2021 | 237,373 ^a^ | 30,423 ^b^ | 14,528 ^e^ | 15,183 ^d^ |
| October | 2021 | 232,962 ^a^ | 29,161 ^b^ | 14,112 ^e^ | 14,728 ^d^ |
| November | 2021 | 241,513 ^a^ | 30,265 ^b^ | 15,363 ^e^ | 14,712 ^d^ |
| December | 2021 | 243,281 ^a^ | 31,924 ^b^ | 15,758 ^e^ | 16,077 ^d^ |
| January | 2022 | 231,045 ^a^ | 28,865 ^b^ | 14,125 ^e^ | 14,500 ^d^ |
| February | 2022 | 227,404 ^a^ | 27,169 ^b^ | 13,106 ^e^ | 13,454 ^d^ |
| March | 2022 | 253,883 ^a^ | 31,533 ^b^ | 14,613 ^e^ | 15,203 ^d^ |
| April | 2022 | 215,856 ^f^ | 28,641 ^b^ | 12,983 ^e^ | 13,842 ^d^ |
| May | 2022 | 231,847 ^f^ | 30,915 ^b^ | 13,958 ^e^ | 14,860 ^d^ |
| June | 2022 | 220,089 ^f^ | 28,828 ^b^ | 13,149 ^e^ | 13,958 ^d^ |
| July | 2022 | 212,621 ^f^ | 26,923 ^b^ | 12,946 ^e^ | 13,169 ^d^ |
| August | 2022 | 211,875 ^f^ | 30,604 ^b^ | 13,549 ^e^ | 13,923 ^d^ |
| September | 2022 | 217,842 ^f^ | 28,259 ^b^ | 13,498 ^e^ | 13,586 ^d^ |
| October | 2022 | 218,862 ^f^ | 28,883 ^b^ | 13,375 ^e^ | 12,886 ^d^ |
| November | 2022 | 225,053 ^f^ | 30,045 ^b^ | 13,409 ^e^ | 13,666 ^d^ |
| December | 2022 | 222,496 ^f^ | 28,330 ^b^ | 13,918 ^e^ | 13,802 ^d^ |
| January | 2023 | 225,995 ^f^ | 27,288 ^b^ | 13,144 ^e^ | 13,372 ^d^ |
| February | 2023 | 206,159 ^f^ | 26,075 ^b^ | 12,166 ^e^ | 11,779 ^d^ |
| March | 2023 | 237,427 ^g^ | 31,019 ^b^ | 13,606 ^e^ | 13,292 ^d^ |
| April | 2023 | 190,719 ^g^ | 27,203 ^b^ | 11,696 ^e^ | 11,634 ^d^ |
| May | 2023 | 209,289 ^g^ | 28,726 ^b^ | 12,133 ^e^ | 12,445 ^d^ |
| June | 2023 | 208,853 ^g^ | 27,611 ^b^ | 11,963 ^e^ | 12,668 ^d^ |
| July | 2023 | 205,092 ^g^ | 27,547 ^b^ | 12,080 ^e^ | 11,707 ^d^ |
| August | 2023 | 202,532 ^g^ | 25,118 ^b^ | 12,515 ^e^ | 12,749 ^d^ |

1. Source: NHS Business Services Authority. Data obtained following a request (FOI-27646) made under the Freedom of Information Act 2000 (ePACT2, NHS Business Services Authority copyright 2022).
2. Source: Public Health Scotland. Data obtained from published dental prescribing abstracts (adapted from public sector information licensed under the Open Government Licence v3.0) [cited 2023 Dec 10]. Available from: <https://www.opendata.nhs.scot/dataset/prescriptions-in-the-community>
3. Source: NHS Wales Shared Services Partnership. Data obtained following a request (6-23) made under the Freedom of Information Act 2000 (NHS Wales Shared Services Partnership copyright 2023).
4. Source: HSC Business Services Organisation. Data obtained from the Family Practitioner Services Pharmacy Payment System (HSC Business Services Organisation copyright 2023).
5. Source: NHS Wales Shared Services Partnership. Data obtained from published dental prescribing abstracts (adapted from public sector information licensed under the Open Government Licence v3.0) [cited 2023 Dec 10]. Available from: <https://nwssp.nhs.wales/ourservices/primary-care-services/general-information/data-and-publications/prescribing-data-extracts/dental-prescribing-data-extract/>
6. Source: NHS Business Services Authority. Data obtained following a request (FOI-01142) made under the Freedom of Information Act 2000 (ePACT2, NHS Business Services Authority copyright 2023).
7. Source: NHS Business Services Authority. Data obtained following a request (FOI-01596) made under the Freedom of Information Act 2000 (ePACT2, NHS Business Services Authority copyright 2023).

**Table S2** Mid-year population estimates or population projections for England, Scotland, Wales, and Northern Ireland between 2016 and 2023.

|  | **Mid-year population estimate or population projection, persons** | | | |
| --- | --- | --- | --- | --- |
| **Year** | **England** | **Scotland** | **Wales** | **Northern**  **Ireland** |
| 2016 | 55,289,034 ^a^ | 5,404,700 ^b^ | 3,077,165 ^a^ | 1,866,042 ^c^ |
| 2017 | 55,619,548 ^a^ | 5,424,800 ^b^ | 3,081,366 ^a^ | 1,875,178 ^c^ |
| 2018 | 55,924,528 ^a^ | 5,438,100 ^b^ | 3,083,840 ^a^ | 1,886,259 ^c^ |
| 2019 | 56,230,056 ^a^ | 5,463,300 ^b^ | 3,087,732 ^a^ | 1,898,519 ^c^ |
| 2020 | 56,325,961 ^a^ | 5,466,000 ^b^ | 3,104,483 ^a^ | 1,900,523 ^c^ |
| 2021 | 56,554,891 ^a^ | 5,479,900 ^b^ | 3,105,633 ^a^ | 1,904,564 ^c^ |
| 2022 | 57,106,398 ^a^ | 5,482,000 ^d^ | 3,131,640 ^a^ | 1,910,543 ^c^ |
| 2023 | 57,535,000 ^d^ | 5,490,000 ^d^ | 3,207,000 ^d^ | 1,910,393 ^e^ |

1. Source: Office for National Statistics (ONS). Estimates of the population for England and Wales [Internet]. Fareham (UK): ONS; 2023 [cited 2023 Dec 10]. Available from: <https://www.ons.gov.uk/peoplepopulationandcommunity/populationandmigration/populationestimates/datasets/estimatesofthepopulationforenglandandwales>
2. Source: National Records of Scotland (NRS). Population estimates time series data [Internet]. Edinburgh (UK): NRS; 2022 [cited 2023 Dec 10]. Available from: <https://www.nrscotland.gov.uk/statistics-and-data/statistics/statistics-by-theme/population/population-estimates/mid-year-population-estimates/population-estimates-time-series-data>
3. Source: Northern Ireland Statistics and Research Agency (NISRA). 2022 mid-year population estimates for Northern Ireland [Internet]. Belfast (UK): NISRA; 2023 [cited 2023 Dec 10]. Available from: <https://www.nisra.gov.uk/publications/2022-mid-year-population-estimates-northern-ireland>
4. Source: ONS. 2020-based interim national population projections: year ending June 2022 estimated international migration variant [Internet]. Fareham (UK): ONS; 2023 [cited 2023 Dec 10]. Available from: <https://www.ons.gov.uk/peoplepopulationandcommunity/populationandmigration/populationprojections/datasets/2020basedinterimnationalpopulationprojectionsyearendingjune2022estimatedinternationalmigrationvariant>
5. Source: NISRA. 2020-based interim population projections for Northern Ireland [Internet]. Belfast (UK): NISRA; 2022 [cited 2023 Dec 10]. Available from: <https://www.nisra.gov.uk/publications/2020-based-interim-population-projections-northern-ireland>

**Table S3** Performance of predictive models for England, Scotland, Wales,

and Northern Ireland between March 2016 and February 2020 assessed using goodness-of-fit criteria.

|  | **England** | **Scotland** | **Wales** | **Northern**  **Ireland** |
| --- | --- | --- | --- | --- |
| MAPE | 3.2 | 3.3 | 3.2 | 4.0 |
| MaxAPE | 10.8 | 10.2 | 9.9 | 11.5 |

Abbreviations: MAPE, mean absolute percentage error; MaxAPE, maximum absolute percentage error.
